# Editorial: Clinical application of multimodal imaging in neuro-ophthalmic diseases

**DOI:** 10.3389/fneur.2025.1591128

**Published:** 2025-04-02

**Authors:** Yuan Liu, Changzheng Chen, Libin Jiang, Hui Yang

**Affiliations:** ^1^Bascom Palmer Eye Institute, University of Miami Health System, Miami, FL, United States; ^2^Renmin Hospital of Wuhan University, Wuhan, Hubei, China; ^3^Beijing Tongren Hospital, Capital Medical University, Beijing, China; ^4^State Key Laboratory of Ophthalmology, Zhongshan Ophthalmic Center, Sun Yat-sen University, Guangzhou, Guangdong, China

**Keywords:** neuro-ophthalmic diseases, multimodal imaging, glaucoma, optic neuritis, hereditary optic neuropathy, optic nerve tumors, ischemic optic neuropathy

## 1 Introduction

The retina and optic nerve, as direct extensions of the central nervous system (CNS), provide a unique window into neurological health (1). Neuro-ophthalmology leverages this relationship to diagnose and manage systemic and neurological disorders that manifest in the visual pathway. Neuro-ophthalmic diseases, with their complex pathophysiology and diverse clinical presentations, demand advanced diagnostic tools (2–4). Recent advancements in multimodal imaging have revolutionized this field, enabling precise visualization of structural and functional changes (5, 6). This editorial highlights two research objectives: (1) pioneering novel imaging technologies to redefine clinical paradigms, and (2) uncovering innovative applications of established techniques to enhance diagnostic and therapeutic precision.

## 2 Pioneering novel imaging technologies

### 2.1 Elevated intracranial pressure: unveiling biomarkers with advanced imaging

Graven-Nielsen et al. harnessed cutting-edge imaging modalities—including en-face OCT, B-scans, adaptive optics scanning light ophthalmoscopy (AOSLO), and fundus photography—to identify peripapillary hyperreflective ovoid mass-like structures (PHOMS), peripapillary wrinkles (PPW), and retinal folds (RF) as biomarkers for elevated intracranial pressure (ICP). Elevated ICP, a life-threatening condition often linked to idiopathic intracranial hypertension or space-occupying lesions, lacks reliable non-invasive diagnostic tools. PHOMS, appearing as hyperreflective ovoid lesions adjacent to the optic disc on OCT, were previously misclassified as variants of optic disc drusen. However, Graven-Nielsen et al. demonstrated their distinct association with axoplasmic stasis secondary to optic nerve head compression. This work positions PHOMS as critical biomarkers for early ICP detection, offering clinicians a non-invasive diagnostic avenue.

### 2.2 Functional imaging in visual field defects: bridging structure and function

Cheng et al. reviewed the role of functional imaging techniques—magnetic resonance imaging (MRI), diffusion tensor imaging (DTI), and optical coherence tomography (OCT)—in diagnosing visual field defects (VFDs). Traditional perimetry identifies functional deficits but provides limited insight into underlying structural pathology. Cheng et al. emphasized how MRI localizes retrochiasmal lesions (e.g., occipital infarcts), DTI quantifies white matter integrity in the optic radiations, and OCT detects retinal ganglion cell layer thinning. This multimodal approach bridges the gap between functional loss and structural damage, enabling targeted interventions for conditions like glaucoma, optic neuritis, and stroke.

### 2.3 COVID-19-associated optic neuritis: imaging a novel entity

The COVID-19 pandemic has unveiled rare but significant neuro-ophthalmic complications. Zhao et al. conducted the first comparative analysis of COVID-19-associated optic neuritis (ON) against classical subtypes, such as neuromyelitis optica (NMO-ON) and myelin oligodendrocyte glycoprotein antibody-associated disease (MOG-ON). Orbital MRI emerged as pivotal, revealing bilateral, long-segment optic nerve enhancement without intracranial lesions—a hallmark of COVID-19 ON. Additionally, universal optic disc edema on fundus imaging and rapid glucocorticoid response suggested an immune-mediated mechanism, likely triggered by molecular mimicry post-infection. This study underscores the role of multimodal imaging in characterizing emerging diseases and guiding therapy.

## 3 Novel applications of established techniques

### 3.1 Differentiating optic neuropathies with OCT angiography

Xiao Q. et al. demonstrated the utility of optical coherence tomography angiography (OCTA) in distinguishing non-arteritic anterior ischemic optic neuropathy (NAION) from demyelinating optic neuritis (DON). By analyzing peripapillary vessel density changes over time, they found that NAION exhibited early, persistent capillary dropout, while DON showed transient reductions with recovery. This differentiation is critical, as NAION requires vascular risk factor management, whereas DON necessitates immunomodulation. OCTA thus serves as a biomarker for early, accurate diagnosis, improving patient outcomes.

### 3.2 Reevaluating PHOMS: a case for OCT's diagnostic superiority

In a separate study, Xiao D. et al. leveraged OCT, OCT angiography (OCTA), and fundus imaging to delineate the characteristics of peripapillary hyperreflective ovoid mass-like structures (PHOMS) across diverse conditions, including tilted disc syndrome, papilledema, and even healthy individuals. Their work emphasized OCT's ability to differentiate PHOMS from optic disc drusen through features like smooth margins and homogeneous hyperreflectivity. OCTA further revealed microvascular changes within PHOMS, suggesting their role as biomarkers of mechanical or inflammatory stress on the optic nerve. This study redefines PHOMS as indicators of axoplasmic stasis rather than drusen variants, highlighting OCT's value in refining neuro-ophthalmic diagnoses.

### 3.3 High myopia and optic nerve head abnormalities: multimodal surveillance

Hu et al. emphasized the role of multimodal imaging—including OCT, OCTA, fundus photography, and indocyanine green angiography (ICGA)—in diagnosing and monitoring optic nerve head (ONH) abnormalities in high myopia. OCT and OCTA provide high-resolution insights into peripapillary atrophy, choroidal thinning, and microvascular dropout, while AI-driven analysis enhances early detection of myopic optic neuropathy. These tools enable personalized interventions to mitigate irreversible vision loss in this growing population.

### 3.4 Migraine and retinal biomarkers: a neurovascular perspective

Chaliha et al. explored the application of OCT and OCTA in migraine, linking retinal thickness and perfusion deficits to aura severity. Focal capillary constriction and ganglion cell layer thinning suggest neurovascular dysregulation, positioning OCTA as a non-invasive biomarker for migraine subtyping and therapeutic monitoring.

## 4 Integrating innovations and envisioning the future

The collective advancements highlighted in this Research Topic underscore the transformative role of multimodal imaging in neuro-ophthalmology. From identifying peripapillary hyperreflective ovoid mass-like structures (PHOMS) as biomarkers of elevated intracranial pressure to characterizing COVID-19-associated optic neuritis through orbital MRI, these studies exemplify how cutting-edge technologies bridge clinical observation and mechanistic insight. Innovations such as OCT angiography (OCTA) have redefined diagnostic paradigms, enabling differentiation of optic neuropathies like NAION and DON, while AI-driven analysis promises to revolutionize early detection of myopic optic neuropathy and migraine-related vascular changes.

Looking ahead, the integration of these tools into cohesive diagnostic frameworks demands three critical steps: (1) Standardization of Protocols: Harmonizing imaging techniques across institutions to ensure reproducibility and comparability of findings. (2) AI and Advanced Analytics: Leveraging machine learning to decode complex imaging patterns, predict disease progression, and personalize therapies. (3) Validation of Biomarkers: Establishing OCTA-derived vascular metrics and PHOMS as prognostic indicators through rigorous longitudinal studies.

The COVID-19 pandemic has further emphasized the need for agility in diagnosing emerging neuro-ophthalmic conditions, where multimodal imaging serves as both a diagnostic anchor and a research catalyst. As these technologies evolve, their translation into routine clinical practice will hinge on interdisciplinary collaboration—uniting ophthalmologists, neurologists, and data scientists to refine precision medicine.

In conclusion, the fusion of novel imaging technologies and repurposed established techniques has irrevocably altered neuro-ophthalmic practice, transforming once-elusive pathologies into actionable insights. The journey forward lies in harnessing these tools not merely to visualize the invisible but to anticipate and intercept disease. By prioritizing innovation, standardization, and collaboration, the field can ensure that the eye remains a pivotal window into neurological health, guiding interventions that preserve vision and improve quality of life for patients worldwide.
